# Defining the phenotype, pathogenesis and treatment of Crohn’s disease associated spondyloarthritis

**DOI:** 10.1007/s00535-020-01692-w

**Published:** 2020-05-04

**Authors:** Anand Kumar, Dana Lukin, Robert Battat, Monica Schwartzman, Lisa A. Mandl, Ellen Scherl, Randy S. Longman

**Affiliations:** 1 Jill Roberts Center for Inflammatory Bowel Disease, Weill Cornell Medicine, 413 E 69th Street, 7th Floor, New York, NY 10021 USA; 2 Hospital for Special Surgery, Weill Cornell Medicine, New York, NY USA

**Keywords:** Crohn’s disease, Spondyloarthritis, Microbiome

## Abstract

Peripheral and axial spondyloarthritis are the most common extra-intestinal manifestations reported in patients with Crohn’s disease. Despite the frequency of Crohn’s disease associated spondyloarthritis, clinical diagnostic tools are variably applied in these cohorts and further characterization with validated spondyloarthritis disease activity indexes are needed. In addition, the pathogenesis of Crohn’s disease associated spondyloarthritis is not well understood. Evidence of shared genetic, cellular, and microbial mechanisms underlying both Crohn’s disease and spondyloarthritis highlight the potential for a distinct clinicopathologic entity. Existing treatment paradigms for Crohn’s disease associated spondyloarthritis focus on symptom control and management of luminal inflammation. A better understanding of the underlying pathogenic mechanisms in Crohn’s disease associated spondyloarthritis and the link between the gut microbiome and systemic immunity will help pave the way for more targeted and effective therapies. This review highlights recent work that has provided a framework for clinical characterization and pathogenesis of Crohn’s disease associated spondyloarthritis and helps identify critical gaps that will help shape treatment paradigms.

## Introduction

Crohn’s disease (CD) has long been recognized to co-exist with systemic inflammation resulting in extra-intestinal manifestations (EIMs). Population studies have suggested that up to 30–40% of patients with active inflammatory bowel disease (IBD) experience EIMs [1, 2]. Arthritis is the most common EIM reported in IBD, which is more prevalent in CD than in ulcerative colitis (UC) [3-5]. In most cases, symptoms of spondyloarthritis (SpA) follow the diagnosis of IBD; however, in nearly 20% cases, joint inflammation precedes initial diagnosis of intestinal disease [6, 7]. Although both peripheral and axial joints can be involved, inflammation of the sacroiliac joints is classically associated with IBD. Despite the frequency of inflammatory joint symptoms, CD-associated seronegative spondyloarthritis (CD-SpA) is often under-diagnosed and inadequately assessed by gastroenterologists [3, 8]. This under-diagnosis  of CD-SpA limits the ability to track symptom response and ultimately impedes delivery and optimization of therapy. Refined clinical diagnostic criteria and disease activity indices have helped guide recent studies in elucidating the pathogenesis of CD-SpA, offering critical insight into treatment selection for this distinct clinicopathologic entity. This review will highlight recent progress in clinical characterization and pathogenesis of CD-SpA, the impact this has on treatment paradigms, and critical gaps that need to be addressed.

## Classifying and characterizing spondyloarthritis in Crohn’s disease

Even early descriptions of CD recognized the co-occurrence of intestinal inflammation with arthritis. These clinical findings of joint inflammation reflect a spectrum of SpA characterized based on the involvement of peripheral or axial joints. Peripheral spondyloarthritis can be pauciarticular (previously called Type I) or polyarticular (previously called Type II) joint inflammation [4, 9, 10]. Axial spondyloarthritis, traditionally referred to as ankylosing spondylitis (AS), includes inflammation of the spine and/or sacroiliac joint. Although earlier literature distinguished clinical SpA based on concordance or discordance with intestinal disease activity, histologic and molecular evidence of subclinical intestinal inflammation support a more inclusive spectrum of disease linking gut and systemic immunity [11, 12]. Reflecting this underlying state of systemic inflammation, spondyloarthritis frequently co-occur with other EIMs including erythema nodosum and uveitis. While this overall description serves as a framework for a spectrum of musculoskeletal symptoms associated with CD, challenges exist in defining both clinical disease burden and underlying pathogenic mechanisms that are needed to guide treatment strategies for CD-SpA.

One challenge in assessing the overall burden of CD-SpA is the lack of uniformity in applying conventional clinical definitions of SpA. Strict characterization of point prevalence of peripheral arthritis in an IBD cohort on physical exam can be very low (< 1%), however inclusion of patient reported self-limited episodes increases the prevalence considerably (up to 12%) [13]. Including patient reported episodes introduces the risk of reporting non-inflammatory arthropathies in these prevalence estimates. As such, the wide variability in prevalence of IBD associated peripheral arthritis (5–44%) and axial inflammation (1–25%) reflect this lack of uniformity in clinical disease characterization [3-5, 13], likely arising from the use of non-standardized disease definitions, not distinguishing non-inflammatory arthralgia from arthritis, and the lack of rheumatologic evaluation. The recent European Crohn’s and Colitis Organization (ECCO) guidelines have also addressed this issue, highlighting a need to ensure accurate identification of true inflammatory arthritis [10]. Criteria established by the Assessment of SpondyloArthritis international Society (ASAS) defines both axial and peripheral SpA. In the context of CD, ASAS criteria define axial SpA as sacroiliitis on magnetic resonance imaging (MRI) or HLA-B27 positivity with one other SpA feature such as inflammatory back pain [14]. Similarly in the context of CD, ASAS criteria define peripheral SpA as the presence of peripheral arthritis, or enthesitis or dactylitis [15] (Table 1). These clinical criteria demonstrate strong sensitivity and specificity for correlation with clinical assessment by rheumatologists [16], but have been applied variably in IBD cohorts.

**Table 1:** Classification criteria for CD-SpA adapted from the Assessment of SpondyloArthritis international Society (ASAS) criteria [14, 15]

Axial CD-SpA	Peripheral CD-SpA
Inflammatory back pain^a^ in a patient with CD AND Sacroiliitis on imaging^b^ OR HLA B-27 antigen positivity	Arthritis and/or dactylitis and/or enthesitis in a patient with CD AND Exclusion of other specific forms of inflammatory joint disease

*CD* Crohn’s disease, *CD-SpA* Crohn’s disease associated spondyloarthritis, *SpA* spondyloarthritis

^a^Insidious onset, chronic back/buttock pain with morning stiffness lasting ≥ 30 min, improvement with activity and nocturnal exacerbation

^b^Active inflammation on MRI highly suggestive of sacroiliitis OR definite radiographic sacroiliitis according to modified New York criteria

Retrospective analysis of longitudinal follow up studies using ASAS criteria to characterize SpA in IBD cohorts provided estimates of axial SpA (7.7–12.3%) and peripheral SpA (9.7–27.9%) [17, 18]. These studies serve as a strong basis for validating the use of modified ASAS guidelines in defining CD-SpA in future research.

In addition, there is a significant unmet need for the uniform application of joint disease activity indices in CD-SpA to establish validity, reliability, and responsiveness for clinical evaluation as well as endpoint assessment in research studies. The Bath Ankylosing Spondylitis Disease Activity Index (BASDAI) is a patient-reported tool that has been clinically validated for assessment of inflammatory activity and response to therapy in both axial and peripheral SpA [19-21]. Ankylosing Spondylitis Disease Activity (ASDAS) includes patient-reported individual and a global activity score and either c-reactive protein (CRP) or erythrocyte sedimentations rate (ESR) [22]. While the inclusion of CRP or ESR provides an objective measurement of inflammatory burden, there are limitations to assessments based on the subjective patient-reported symptom scores. Although BASDAI and ASDAS have also been used to assess arthritis activity and response to treatment in IBD-related SpA [23, 24], these scores do not always correlate with joint inflammatory activity in IBD [25, 26]. Additionally, these scores are validated mostly in axial disease and while they may similarly provide an accurate measure of peripheral disease, patients with predominantly peripheral SpA may benefit from a more focused evaluation [27]. Peripheral joint characterization included in more extensive exams including Peripheral SpA Response Criteria (PSpARC40) may more accurately assess response, but the required joint examinations by an expert rheumatologist make the broader use of these instruments in gastroenterology practices less practical [28]. Finally, MRI has revolutionized assessment of SpA over the last two decades, but no validated criteria have yet been developed to assess disease severity or response to therapy in IBD. Thus, there remains a need for studies to validate these indices in CD-SpA and to correlate with pathogenic biomarkers to help guide therapy.

## Elucidating the pathogenesis of spondyloarthritis in Crohn’s disease

**Fig. 1:**
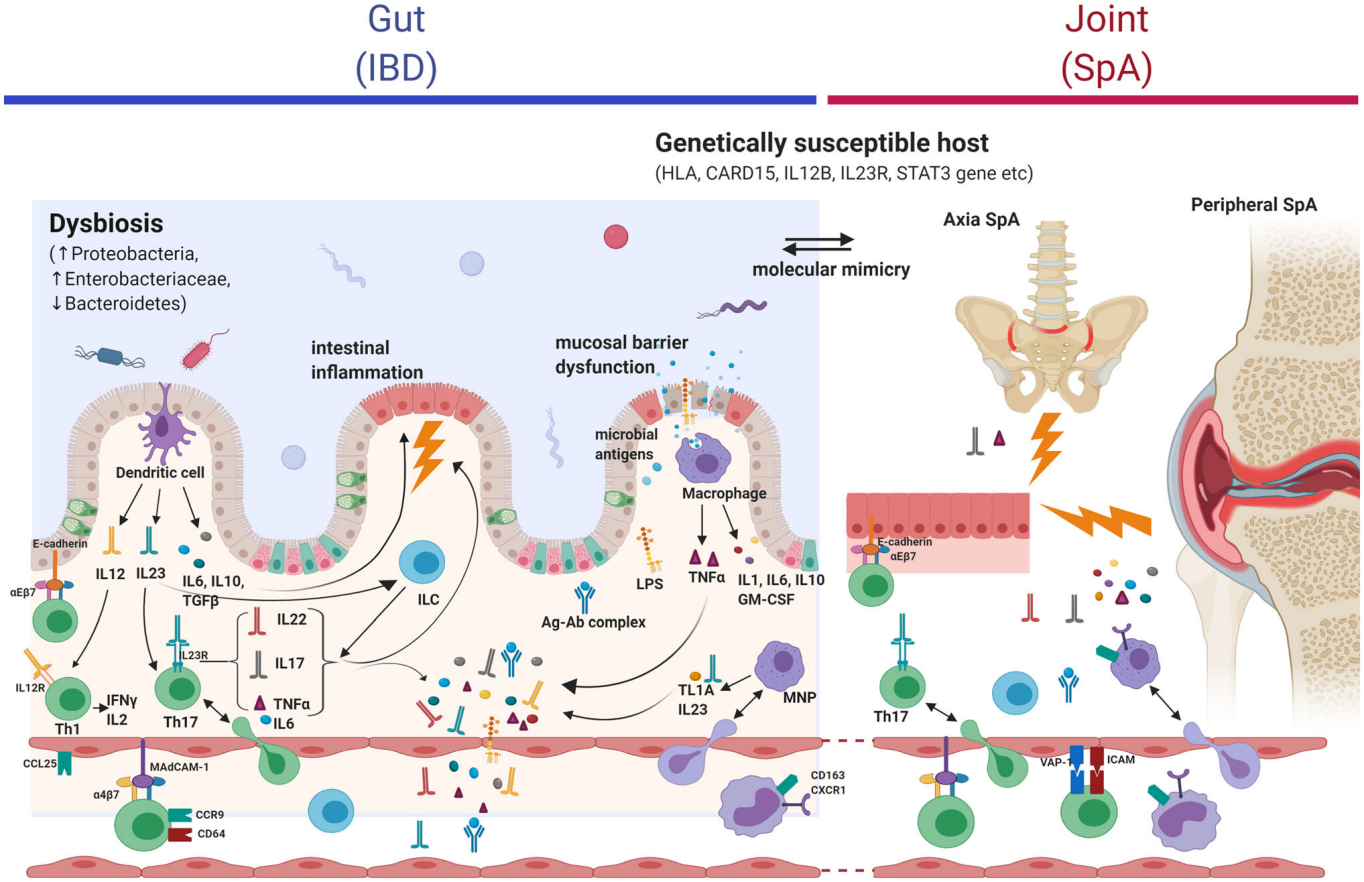
Pathogenic mechanisms of Crohn’s-associated spondyloarthritis. *Ag-Ab* antigen–antibody complex, *CARD15* Caspase recruitment domain-containing protein 15, *CD* Crohn’s disease, *CD-SpA* Crohn’s disease associated spondyloarthritis, *GM-CSF* granulocyte monocyte colony stimulating factor,* HLA* human leukocyte antigen, *IBD* inflammatory bowel disease, *IL* Interleukin, *ILC* innate lymphoid cell, *IFN* interferon, *LPS* lipopolysaccharide, *MNP* mono-nuclear phagocytes, *SpA* spondyloarthritis, *TNF* tumor necrosis factor, *Th* helper T cells. Genetic susceptibility: presence of HLA genes (B27, B35, B44) and polymorphisms in IL-23R, IL-12B, STAT3, and CARD9, CARD15 genes increase the susceptibility of host to both IBD and SpA. Intestinal dysbiosis: abundance of *Proteobacteria, Enterobacteriaceae, Ruminococcus gnavus* and a reduction in Bacteroidetes in patients with CD-SpA. Additionally, abundance of *Dialister, R. gnavus, Prevotella* (axial SpA) seen in SpA. Molecular mimicry: cross reactivity between peptide sequences common between enteric bacteria and host HLA. Autoimmune gut inflammation in CD triggered by genetic and environmental factors leading to activation of IL23-IL17 axis and intestinal phagocytic cells. Extension of immune response from gut to joint could occur through: Molecular mimicry; Trafficking of activated immune cells (T cells, macrophages, innate lymphoid cells) facilitated by: ectopic expression of gut-specific chemokines (CCL25), adhesion molecules (MAdCAM, ICAM, E-cadherin) and integrins (α4β7, αEβ7) in the joint; binding to non-gut-specific adhesion molecules (VAP-1) and chemokine receptors (CXCR3, CCR5); Intestinal mucosal barrier dysfunction and translocation of microbial antigens/products (LPS); Increased inflammatory mediators and proinflammatory cytokines (IL-6, TNFα, IFNγ,) in serum; Circulating autoantibodies with epitopes shared between the gut and joint

The pathogenesis of CD-SpA remains poorly understood. A variety of pathogenic mechanisms have been proposed including those which result from an extension of gut-specific inflammatory processes as well as non-specific alterations in the systemic inflammatory milieu [10] (Fig. 1). The strongest genetic susceptibility to SpA lies within the major histocompatibility complex (MHC) class I locus with human leucocyte antigen gene (HLA)-B27 conferring the highest genetic risk association to date [29]. Genetic risk variants individually associated with either SpA or IBD overlap significantly in the interleukin (IL) 23-IL17 pathway, although no specific genetic markers of IBD-associated SpA have been defined [30]. These findings highlight the likely interaction of multiple genetic pathways as well as the potential role for environmental and/or microbial factors, which synergistically or independently act to modulate inflammation in a genetically susceptible host. Here, we will focus on the IL23-IL17 pathway and its potential intersection with the gut microbiome.

### Elucidating the cellular mechanism for IL-23 dependent inflammation in CD-SpA

Early studies demonstrated that susceptibility variants in the IL23R locus were associated with IBD and SpA independently [31]. These studies initially postulated a role of IL-23 in supporting IL-17 producing Th17 CD4+ T cells [32]. IL-17 as an effector molecule can play a key role in neutrophil recruitment and sustaining the inflammatory environment. Supporting the importance of IL-17, analysis of both synovial tissue and circulating blood in patients with inflammatory arthritis or AS showed an upregulation of Th17 cells which positively correlated with joint disease activity scores [33-36]. Linkage of susceptibility variants in the IL17 pathway (RUNX3, IL23R, IL6R, IL1R2, IL12B, TYK2) [37] further support the potential pathogenic role for this subset. Despite initial reports of molecular mimicry as the mechanism underlying HLA-B27 dependent disease [38], more recent work showed that misfolding of HLA-B27 can lead to increased IL-23 and enable HLA-B27 homodimers to bind KIR3DL2 on IL-17+ CD4+ cells in blood and synovium [39-41]. These findings link the strong HLA-B27 association seen in AS with the IL-23-Th17 pathway [42].

Despite the initial focus on Th17 immunity, other cells in addition to CD4+ T cells were found to be predominant producers of IL-17. CD8+ T cells in particular were shown to have higher IL-17 production in the peripheral blood and synovial joint in psoriatic arthritis (PsA), which is a type of SpA affecting primarily the peripheral joints [43]. Of note, this finding was seen in SpA, but not in rheumatoid arthritis (RA), reflecting the specific association of SpA with MHC class I alleles. Experimental models of enthesitis further highlighted the role of non-conventional IL-23R responsive T cells [44], but their potential role in human disease is still not clear.

Although IL-17 is a main effector cytokine downstream of IL-23, other cell types as well as effector molecules exist in this pathway, demonstrating the complexity of their potential contribution to SpA. In particular, the discovery of group 3 innate lymphoid cells (ILCs) defined a unique innate cell type capable of producing IL-22 and GM-CSF in response to IL-23 [45]. The discovery of ILCs coupled with reports of discordance between IL23A and IL17 in the ileal tissue, raised this possibility that IL-23 responsive ILCs may play a critical role in SpA independent of IL17 [46]. Intestinal CX3CR1+ mononuclear phagocytes (MNPs) are a main source of IL-23 that activate ILCs, and these MNPs are increased in patients with AS [47]. In addition, MNP production of tumor necrosis factor (TNF)-like cytokine 1A (TL1A), which is increased in the synovium of patients with AS, synergizes with IL-23 to regulate the production of IL-22 [48]. These finding highlight an array of innate effectors linking IL-23 with gut-joint inflammation.

Thus, although these findings highlight a strong genetic basis for the IL-23 pathway in CD-SpA, the cellular mechanism and key effectors molecules still need to be clarified. With the development of biologic therapies targeting specific aspects of these pathways, a mechanistic understanding will be essential to developing strategic treatment paradigms in CD-SpA. Furthermore, given the overlap of genetic risk in CD and SpA, it will be important to understand the underlying intestinal triggers of SpA.

### Understanding the potential role for the microbiome in CD-SpA

Even before the discovery of the IL-23 dependence of disease pathogenesis, the gut was recognized as a potential portal for SpA initiation. Subclinical luminal inflammation is seen in 60% of patients with SpA and defined by key histologic features in SpA [46, 49]. Fecal calprotectin is a promising strategy to identify these patients [50]. This led to the hypothesis that opportunistic pathobionts in the gut could potentially trigger an immune response at a distant extra-intestinal site in a genetically predisposed host. Since then, several studies have been conducted to explain the mechanisms that could help explain this phenomenon.

The microbiome was viewed as an early potential trigger for seronegative SpA, partly due to the association of enteric pathogens such as *Salmonella*, *Campylobacter*, *Yersinia* and *Shigella* in reactive arthritis. Early clinical data revealed a potential association between fecal *Klebsiella* and AS [51]. Cross-reactivity of HLA-B27 with *Klebsiella* supported a model of molecular mimicry in which the amino acid sequence, QTDRED, present in HLA-B27 was found to have similarity with nitrogenase reductase enzyme in *Klebsiella* [38]. This link was further supported by studies of HLA-B27 transgenic rats which when re-derived under germ-free conditions showed attenuation in AS [52, 53]. Although subsequent clinical studies did not uniformly support this association of SpA with *Klebsiella*, more recent studies using high throughput sequencing technology have allowed for more complete microbiome analysis. In particular, the mucosal-associated microbiome from ileal biopsy of patients with new onset treatment- naïve SpA (without IBD) showed a linear correlation between the abundance of the genus *Dialister* with both clinical disease activity (measured by ASDAS) and joint tissue inflammation [54]. Additional alterations have been noted in the fecal microbiota of patients with SpA, most notably the increase in *Ruminococcus gnavus* [55]. These changes also correlate with clinical disease activity (measured by BASDAI). Although intestinal inflammation was not assessed, the abundance of *R. gnavus* was also higher in the subset of patients with IBD-associated SpA. The expansion of *Ruminococcus* may be unique to axial SpA as cohort analysis of psoriatic arthritis, in contrast, showed a reduction in *Ruminococcus* and *Akkermansia* [56]; however, medication treatment of psoriatic arthritis with non-selective anti-inflammatory drugs (NSAIDs) or methotrexate could have impacted these findings. Large metagenomic analysis of 211 participants from China demonstrated significant alterations in the microbiome in patients with AS characterized by an increase in *Prevotella* species [57]. The increase in *Prevotella copri* also seen in RA may reflect a shared mechanism underlying joint inflammation [58]. Similarly, an increased abundance of *Clostridiaceae* was shared by both IBD-associated arthropathy and RA patients and suggests a potentially common microbial link for inflammatory arthritis [59]. Collectively, these studies support a correlation between the gut microbiome and axial SpA, but further studies are needed to assess the impact of geography, diet, and medication in the diagnostic and therapeutic potential of these biomarkers.

Several recent studies have revealed potential mechanisms of microbial impact on systemic inflammation. For example, strain specific lipoglycans from *R. gnavus* have been shown to act as potent antigens with both diagnostic and pathogenic implications for systemic inflammatory disease [60], highlighting the need for strain level characterization in further defining this clinical association. In addition, the functional characteristics of the microbial taxa in the intestinal environment may dictate immune outcomes. Rat models of HLA-B27 disease show a significantly higher level of *Akkermansia* colonization and increased frequency of IgA coating of fecal bacteria with disease activity [61]. These findings illustrate a non-specific contribution of a mucinophilic pathobiont which may correlate HLA type with increased inflammatory tone of Th17 signatures in shared genetic risk pathways [62]. Moreover, the increased abundance of both *E. coli* and *Prevotella* in ileal samples from AS mechanistically regulated zonulin and epithelial permeability [63]. The community effect of this dysbiosis, therefore, potentiates the impact of opportunistic pathobionts.

We have recently evaluated the contribution of the microbiome in IBD-associated peripheral SpA [20]. Using fecal samples from 59 IBD patients with or without SpA defined by ASAS criteria, our results revealed an increased abundance of *Proteobacteria*, specifically *Enterobacteriaceae*, and a reduction in *Bacteroidetes* in patients with CD-SpA compared to active CD alone. Moreover, the abundance of *Enterobacteriaceae* linearly correlated with SpA activity measured using BASDAI. No differences were observed between UC-associated SpA and UC alone suggesting differential contribution of either genetic or microbial pathways specific to CD. To further define immunologically relevant strains, we performed IgA-Seq (or Bug-FACS) in which IgA coated microbes were sorted and sequenced. Analysis of clonal isolates revealed the expansion of adherent-invasive *E. coli* (AIEC) in the IgA-coated fraction of patients with CD-SpA compared to CD alone. These isolates are characterized by their ability to attach to mucosal surfaces and stimulate immune responses. AIEC-specific IgG in the serum was higher in patients with CD-SpA compared to CD supporting systemic impact of particular pathobionts [20]. However, further characterization is needed to understand the role for direct microbial translocation or local immune cell activation that triggers systemic joint inflammation.

An important issue arising from the potential contribution of the microbiome in immune regulation is the role for strain level differences. For *R. gnavus*, overall abundance in the intestine correlated with serum antibodies to only 1/8 *R. gnavus* strains tested [60]. *Akkermansia muciniphila* is a robust inducer of T follicular helper cells and systemic IgG in mouse models, but the potential contribution of strain variability is not known [64]. AIEC contains multiple virulence associated factors which may contribute, including the long polar fimbriae that is associated with mucosal attachment and may regulate induction of cytokines by macrophages [65]. In addition, utilization of propanediol as a carbon source by AIEC impacts mucosal attachment and macrophage survival [66] and isolates capable of this are expanded in CD-SpA, but the contribution of this metabolic function to local and systemic immunity is not well known. These and other strain specific factors may help reveal diagnostic and therapeutic microbial targets of systemic immunity in CD-SpA.

Finally, the impact of non-bacterial constituents of the microbiome including the mycobiome and virome in IBD-associated SpA is not well studied. Fungal dysbiosis in CD is characterized by restricted overall diversity and an expansion in *Candida *spp. [67]. Circulating anti-*S. cerevisiae* antibody levels are elevated in patients with CD, but the pathogenic contribution to either intestinal or systemic inflammation is not clear. Fungal elements such as curdlan are critical in regulating systemic inflammation in animal models of arthritis [68]. A recent study in patients with AS suggest higher levels of *Ascomycota* and decreased abundance of *Basidiomycota*, but the sample size was limited [69] and more studies are needed to assess a functional contribution. Similarly, our understanding of the viral contribution is limited. An expansion of viral like particles (VLP) or bacteriophage occurs during active IBD [70]. In pre-clinical models, these VLPs may directly activate the immune response and lower VLP levels correlate with clinical response to fecal transplant in ulcerative colitis [71], but the impact of the intestinal virome in IBD-associated SpA needs to be investigated in clinically phenotyped cohorts. Further studies elucidating the potential contribution of various microbiome-associated pathways in IBD-associated SpA will help to guide development of diagnostic and therapeutic targets.

## Treatment paradigms for CD-SpA

The progress reviewed above in establishing clinical criteria and elucidating a mechanistic understanding of the pathogenesis of CD-SpA may allow for rational selection of treatment. Besides the gold standard tumor necrosis factor α (TNF) antagonist therapy for SpA, recent commercialization of other biologic therapies targeting α4β7-mediated trafficking of lymphocytes (vedolizumab) and the p40 subunit of IL12/23 (ustekinumab) may help personalize treatment of CD-SpA. However, since there is little direct evidence to support the efficacy of these agents in co-existing IBD and SpA, current treatment strategies are extrapolated from independent studies in SpA [such as AS (axial) and PsA (primarily peripheral SpA)] and IBD or from limited data from post hoc analysis of therapeutic drug trials in IBD (Table 2).

**Table 2:** Pharmacologic treatments for CD-SpA

Axial CD-SpA	Peripheral CD-SpA
Currently approved therapies	
Short term NSAIDs if CD is in remission^a^	Short term NSAIDs if CD is in remission^a^
Anti-TNFα therapy: Infliximab Adalimumab Certolizumab pegol	Local steroid injection for pauciarticular or short-term oral steroid for polyarticular peripheral SpA
	Sulfasalazine
	Methotrexate
	Anti-TNFα therapy: Infliximab, adalimumab, certolizumab pegol
No IL12/23 inhibitor therapy is proven beneficial in axial SpA	Anti IL12/23: Ustekinumab
Other therapies under investigation/development	
Selective JAK-1 inhibitor: Upadacitinib and filgotinib (successful phase 2 trials in AS, PsA and CD; positive results in phase 3 trial of upadacitinib in PsA. Ongoing phase 3 trial in CD)	
α4β7 anti-integrin: vedolizumab Approved for treatment of moderate-to-severe CD. Based on recent a systematic review, may be effective in preventing onset of arthritis in CD, however, may not be effective in improving co-existing arthritis	
Therapies under investigation: S1P1 receptor modulator—ozanimod, etrasimod Anti-TNF like cytokine 1A (anti-TL1A) therapy Fecal microbiota transplant Combination biologic therapy (combining two or more biologics with different mechanism of action)	

*CD* Crohn’s disease, *CD-SpA* Crohn’s disease associated spondyloarthritis, *IL* interleukin, *JAK* Janus kinase, *PsA* psoriatic arthritis, *S1P1* Sphingosine 1-phosphate-1, *SpA* spondyloarthritis, *TNFα* tumor necrosis factor-alpha

^a^Typically < 15 days and should be avoided in active IBD

Non-selective anti-inflammatory drugs (NSAIDs) are the first line of therapy in patients with SpA. While non-selective cyclo-oxygenase (COX) inhibitors are generally contraindicated in patients with IBD due the risk of disease exacerbation [72], two clinical trials found selective COX-2 inhibitors to be safe and effective in the short-term treatment of inactive IBD [73, 74]. While routine NSAID therapy is generally avoided in IBD patients due to concern for potential deleterious effects on the gut mucosa [75], there is growing evidence that COX-2 inhibitors, and specifically celecoxib, are safe and effective.

Sulfasalazine (SAS) is one of the disease modifying drugs effective in treating peripheral arthritis symptoms of IBD, however is not effective in axial-SpA [76]. Although one of the oldest medicines used for the treatment of CD, the mechanism of SAS’s effect is not clear. SAS is a pro-drug that is cleaved by azo-reductase produced by the colonic microbiota into sulfapyridine and 5-aminosalicylate (5-ASA) released into the distal intestine. It remains unclear if there is a role of one or more gut microbe in this activation process of SAS. While 5-ASA may offer the mechanistic benefit to mucosal healing acting through PPARγ (peroxisome proliferator activated receptor gamma) [77, 78], sulfapyridine inhibits bacterial folic acid synthesis. The specific target of sulfapyridine’s anti-bacterial effect in the microbiome is not known. In addition to understanding the therapeutic mechanism of its impact, another consideration is variability in dosing. While rheumatologists frequently treat with  2 g daily, the gastroenterology literature supports the use of 4–6 g daily for active symptoms [79].

TNF antagonist therapy has been a mainstay of therapy both for SpA and CD. The efficacy of TNFα blockade underlies the likely pleiotropic effect of TNFα on many shared pathways. Indeed, the efficacy of anti-TNFα therapy in AS correlates with the reduction of Th17 cells in the peripheral blood following treatment [34]. Despite this response in the peripheral blood, it is not clear that this reflects tissue activity of TNF antagonists in IBD-associated SpA. Systematic reviews of TNF antagonist therapy support the general efficacy of TNFα blockade in IBD-associated EIMs [80, 81], including axial and peripheral SpA. However, the interventional studies that reported efficacy of infliximab, adalimumab and certolizumab pegol in the treatment of CD-associated arthritis did not utilize objective criteria for diagnosis (e.g. ASAS) or to define response/ remission of joint disease (e.g. ASDAS) [80, 82]. While limited study data are available to characterize the impact of joint disease activity [23, 83], these biologics are considered first line therapy for IBD-associated SpA.

Vedolizumab is approved for the induction and maintenance of remission in UC and CD. Although initially discovered and marketed for gut selectivity, vedolizumab (anti-α4β7) may also impact systemic immunity. One theory is that blocking the homing of α4β7 into the tissue increases the abundance of these effectors in the peripheral blood, thereby enhancing systemic immune activation. In one study, HLA-B27 negative de novo or flare of inactive SpA occurred in 11 patients achieving remission of luminal symptoms with vedolizumab [84]. Moreover, compared to patients receiving anti-TNF therapies, IBD patients receiving vedolizumab were more likely to develop EIMs including arthropathy [85]. Despite these findings, post-hoc analysis of the GEMINI trials revealed reduced incidence of arthritis in CD (HR 0.14, 95% CI 0.05–0.35) and no increase in the incidence of arthritis in UC patients treated with vedolizumab versus placebo [86]. Similarly, in the OBSERV-IBD cohort, extra-intestinal inflammation including joint inflammation improved in parallel with bowel disease, however 13% of patients without EIMs at baseline developed non-inflammatory arthralgia [87]. More recently, a systematic review of 11 studies where vedolizumab was used for EIMs in IBD, demonstrated no effect on pre-existing arthralgia and arthritis, and lower incidence of new rheumatic symptoms among vedolizumab users compared to placebo [88]. While there is a signal for benefit of vedolizumab in CD-SpA, a significant limitation of these analyses is the lack of uniform clinical phenotyping of inflammatory arthritis and prospective assignment of disease activity indices, which will need to be addressed in future analyses.

The strong genetic association of both SpA and IBD with IL23R genetic variants and overlapping mechanisms involving the IL23/IL17 axis highlighted the potential role of specific blockade of this pathway in drug development. In addition, elevation of IL-17, but not TNFα, has been reported in CD-SpA compared to active CD alone [20]. However, the translatability of these findings into therapeutic strategies have so far been puzzling. Despite the efficacy of IL-17A inhibitor therapy, secukinumab, for the treatment of AS [89], it was not effective in the treatment of CD and may lead to a higher rate of adverse events, including infections [90, 91]. Recent results in psoriatic arthritis reveal that IL-17A blockade correlated with expansion of *Candida albicans* with features of subclinical gut inflammation [92]. Another potential explanation of this paradox comes from murine models where the suppression of IL-17F, but not of IL-17A, provided protection against colitis by inducing Treg cells through modification of the intestinal microbiota [93, 94]. It is postulated that low levels of IL-17 can be produced from ILCs, Tγδ, independent of IL-23 [95]. The homeostatic role for this IL-23 independent IL-17 may reflect the unexpected results of IL-17 blockade in CD.

In contrast to IL-17 blockade, anti-IL12/23 therapy, ustekinumab is effective in the treatment of patients with Crohn’s disease [96]. Ustekinumab is effective in patients that have failed TNF antagonist therapy suggesting the possibility of a distinct pathway of disease. It is not known if this distinct pathway enriched in patients with CD-SpA. Similarly, selective IL23 antagonist, risankizumab is in development (ongoing phase 3 trials after successful phase 2 trials) for treatment of moderately-to-severely active CD [97, 98]. Although ustekinumab is approved for psoriatic arthritis and was found to be effective in treating concomitant PsA in IBD patients, suggesting its benefit for CD-related peripheral SpA [99, 100], both ustekinumab and risankizumab failed to demonstrate efficacy for AS [101, 102]. Moreover, the switch from TNFα blockade to anti-IL-12/23 blockade has been associated with the unmasking of psoriatic arthritis in a small case series within the psoriasis population [103]. Further studies are needed to assess the impact of these pathways specifically in patients with CD-SpA and tracked with meaningful disease activity markers.

In addition to biologic therapy, small molecule inhibitors of immune pathway are emerging as therapy for both SpA and CD. In particular, the non-selective Janus kinase (JAK) inhibitor, tofacitinib is approved for the treatment of UC, RA and PsA [104-106] and a phase 3 trial in AS is ongoing. While tofacitinib did not meet efficacy endpoints in the phase 2 trials of Crohn’s disease [107], there was a significant biochemical response and evidence from post-hoc analyses suggest benefit in CD as well [108, 109]. In initial trials, selective JAK-1 (filgotinib and upadacitinib) and tyrosine kinase (TYK) inhibitors have shown efficacy in Crohn’s disease [110]. Moreover, both filgotinib and upadacitinib were found to be safe and effective in AS and PsA in their respective phase 2 trials, while upadacitinib met efficacy endpoints in a phase 3 study in PsA [111-113]. These highly encouraging results support enthusiasm for specifically evaluating their efficacy in CD-SpA.

Despite similarities in pathogenesis, CD and SpA maintain unique aspects of disease, which may need to be therapeutically targeted independently. While both entities may co-exist in a patient, it is possible that they do not respond to common therapies and therefore strategies for combining therapies need to be evaluated. Anecdotal cases have raised the possibility of combining anti-α4β7 and anti-TNFα blockade [108, 114]. Therapies directed towards novel mechanistic targets are being studied which also offer great potential. Sphingosine 1-phosphate (S1P) modulation is one such strategy to block T cell egress from lymph nodes into the circulation. In contrast to α4β7 blockade, this strategy may be able to block both tissue and systemic inflammatory symptoms that depend on circulating lymphocytes [115]. In addition, anti-TL1A therapy is in development for treatment of UC. Given the potential role for TL1A in joint inflammation discussed above [46], it remains to be evaluated whether this mechanism would be effective in SpA as well. Finally, fecal microbiota transplant (FMT) is under active investigation for the treatment of IBD with preliminary success in the treatment of UC. The impact of FMT on inflammatory arthritis is unknown. Given the emerging knowledge of the potential role of strain specific pathobionts in IBD-associated SpA, microbial-based therapies may play an important role in modifying clinical outcomes.

## Conclusion

CD-SpA is a unique subset of CD with distinct clinical, cellular, and microbial characteristics. Additional data are needed using ASAS clinical diagnostic criteria and validated joint disease activity indexes to define the clinical burden and therapeutic responses in this unique subset. Pre-clinical and clinical data defining the link between the gut microbiome and the inflammatory immune response in CD-SpA has highlighted key pathways that can be therapeutically targeted. Combined with a multi-disciplinary care team approach, future studies will help guide rational selection of targeted therapies based on disease phenotype and develop a precision medicine approach to spondyloarthritis in Crohn’s disease.

## Abbreviations

5ASA: 5-Aminosalicylate
anti-TL1A: Anti-tumor necrosis factor-like cytokine 1A
AS: Ankylosing spondylitis
ASAS: Assessment of SpondyloArthritis international Society
ASDAS: Ankylosing Spondylitis Disease Activity
BASDAI: Bath Ankylosing Spondylitis Disease Activity Index
CD: Crohn’s disease
CD-SpA: CD-associated spondyloarthritis
CD4: Cluster of differentiation-4
COX: Cyclo-oxygenase
CRP: C-reactive protein
CX3CR1: C-X3-C motif chemokine receptor
EIM: Extra-intestinal manifestation
GM-CSF: Granulocyte monocyte-colony stimulating factor
HLA: Human leucocyte antigen
IBD: Inflammatory bowel disease
Ig: Immunoglobulin
IL: Interleukin
ILC: Innate lymphoid cells
JAK: Janus Kinase
KIR3DL2: Killer cell immunoglobulin-like receptor 3DL2
MHC: Major histocompatibility complex
MNP: Mononuclear phagocytes
MRI: Magnetic resonance imaging
NSAID: Non-selective anti-inflammatory drugs
PsA: Psoriatic arthritis
PPARg: Peroxisome proliferator activated receptor gamma
PSpARC: Peripheral SpA response criteria
SAS: Sulfasalazine
SpA: Spondyloarthritis
RA: Rheumatoid arthritis
Th: T helper cells
TNF: Tumor necrosis factor
Treg: Regulatory T cells
TYK2: Tyrosine kinase 2
UC: Ulcerative colitis
VLP: Viral like particles
